# In Reply to: Fine Needle Aspiration

**DOI:** 10.5812/traumamon.17044

**Published:** 2014-01-25

**Authors:** Ali-Reza Ehsanbakhsh, Amin Saburi

**Affiliations:** 1 Department of Radiology, Faculty of Medicine, Birjand University of Medical Sciences, Birjand, IR Iran; 2 Health Research Center, Baqiyatallah University of Medical Sciences, Tehran, IR Iran

**Keywords:** Diagnosis, Biopsy, Fine-Needle, Sensitivity and Specificity

*Dear Editor,*

In reply to the Letter to the Editor by Prof. Wiwanitki (1) concerning my recent study, we stated that “FNA is a useful atraumatic diagnostic technique with a high diagnostic accuracy which can provide a highly sensitive diagnosis with low false positive diagnoses in patients with nonthyroidal masses” (2). The author stated that FNA has 10.4% false negative results according to a recent study by Wharry et al.; however, this study was performed on thyroidal neck masses, while our study was conducted on nonthyroidal neck masses (3). As we demonstrated in Table 4 of the referred paper, we can see that the sensitivity and specificity of Fine Needle Aspiration (FNA) to diagnosis neck masses is high enough to consider it during the diagnostic process. Moreover, as we mentioned in our paper, the diagnostic properties of FNA depend on the properties of the mass. In masses suspicious for malignancy (such as larger masses, rapidly enlarged masses, masses in patients with history of malignancy or radiation, etc.) the cytology results and the FNA specimens should be more carefully interpreted (2). Also, as a limitation of our study we explained that pathologist experience can affect the cytology results. At our medical center, as a referral center in Iran, there is a group of pathologists who interpret the cytology results (4). There are two interesting comments that merit mentioning; the first is the rate of complications. Although many studies such as a review by Wu and Burstein declared that “FNA is used as one of the most cost-effective, complication-free, and rapid techniques for preoperative investigation of tumors and tumor-like conditions” (5, 6). Secondly, the role of radiologic modalities such as ultrasonography (US) in this paper is less discussed. Almost all studies conducted on the diagnostic accuracy of FNA, confirmed the role of Ultrasounds Imaging (US) to prepare a more useful specimen. Krishnappa et al. stated that “US-guided FNA provides a better representative sample and has a higher diagnostic rate in evaluation of thyroid lesions” (7). Also, some studies demonstrated that “the absence of suspicious US features did not reliably exclude malignancy”; others confirmed the usefulness of radiological properties to select a better site for FNA (8). For example, Moon et al. stated that “A taller-than-wide shape in either the transverse or longitudinal plane, was more accurate and sensitive in predicting thyroid malignancy” (9). Moreover, the size of the nodule is one of the most important US characteristics of a neck mass. There is a wide variety of size cut-off (between 1 to 4 cm) for neck masses suspicious for malignancy (3, 10). Moreover, US as a useful and safe radiologic modality can help find the largest, heterogeneous and more suspicious nodule for FNA.
